# Zinc Supplementation Modulates NETs Release and Neutrophils’ Degranulation

**DOI:** 10.3390/nu13010051

**Published:** 2020-12-26

**Authors:** Weronika Kuźmicka, Aneta Manda-Handzlik, Adrianna Cieloch, Agnieszka Mroczek, Urszula Demkow, Małgorzata Wachowska, Olga Ciepiela

**Affiliations:** 1 Postgraduate School of Molecular Medicine, Medical University of Warsaw, Zwirki i Wigury 61 Street, 02-091 Warsaw, Poland; weronika.kuzmicka@wum.edu.pl; 2 Department of Laboratory Diagnostics and Clinical Immunology of Developmental Age, Medical University of Warsaw, Zwirki i Wigury 63a Street, 02-091 Warsaw, Poland; aneta.manda-handzlik@wum.edu.pl (A.M.-H.); agnieszka.mroczek@wum.edu.pl (A.M.); urszula.demkow@uckwum.pl (U.D.); adrianna.cieloch@wum.edu.pl (A.C.); 3 Doctoral School, Medical University of Warsaw, Zwirki i Wigury 63 Street, 02-091 Warsaw, Poland; 4 Department of Laboratory Medicine, Medical University of Warsaw, Banacha 1a Street, 02-097 Warsaw, Poland

**Keywords:** zinc, neutrophils, neutrophil extracellular traps, neutrophils function, degranulation

## Abstract

Zinc plays an important physiological role in the entire body, especially in the immune system. It is one of the most abundant microelements in our organism and an essential component of enzymes and antibacterial proteins. Zinc levels were reported to be correlated with the intensity of innate immunity responses, especially those triggered by neutrophils. However, as the results are fragmentary, the phenomenon is still not fully understood and requires further research. In this study, we aimed to perform a comprehensive assessment and study the impact of zinc on several basic neutrophils’ functions in various experimental setups. Human and murine neutrophils were preincubated in vitro with zinc, and then phagocytosis, oxidative burst, degranulation and release of neutrophil extracellular traps (NETs) were analyzed. Moreover, a murine model of zinc deficiency and zinc supplementation was introduced in the study and the functions of isolated cells were thoroughly studied. We showed that zinc inhibits NETs release as well as degranulation in both human and murine neutrophils. Our study revealed that zinc decreases NETs release by inhibiting citrullination of histone H3. On the other hand, studies performed in zinc-deficient mice demonstrated that low zinc levels result in increased release of NETs and enhanced neutrophils degranulation. Overall, it was shown that zinc affects neutrophils’ functions in vivo and in vitro. Proper zinc level is necessary to maintain efficient functioning of the innate immune response.

## 1. Introduction

Zinc is the second most abundant intracellular metal in the human body. In the cell, half of the zinc is located in the cytoplasm, whereas 30–40% in the nucleus, and about 10% in the cell membrane [1]. In developed countries, zinc deficiency often affects pregnant women, children, the elderly, vegetarians, and patients suffering from intestinal diseases [2]. In addition, diminished zinc concentration was observed in patients suffering from diabetes mellitus type 2 and in patients with chronic kidney disease. Strikingly, lowered zinc levels have been correlated with an increased risk of breast cancer [3]. On the cellular level, zinc deficiency impairs macrophages’ and neutrophils’ ability to phagocyte and inhibits chemotaxis, NK cell activity and production of reactive oxygen species [2,4,5,6,7]. Zinc affects apoptosis and gene transcription, and its lack in the diet leads to atrophy of the thymus in mice [8]. Importantly, zinc ions are also an essential component of a large number of enzymes and play an important role in the growth and differentiation of cells of the immune system as well as the gastrointestinal tract [9]. Zinc supplementation helps to reduce the incidence of infections, pneumonia and helps to prevent dementia, Alzheimer’s disease, and atherosclerosis [10]. Moreover, there are data showing that zinc supplementation during antibiotic therapy reduces mortality by almost half in newborns suffering from sepsis [11]. On the other hand, reduced resistance to infection was observed in patients during parenteral alimentation without zinc supplementation [12].

Neutrophils are specialized cells of the immune system that help to defend the body against invading microbes. Thanks to their ability to respond immediately to the presence of pathogens, they are essential for the proper functioning of the innate immune response. They are the first cells to reach the site of infection, where they directly eliminate invaders through various strategies such as phagocytosis, degranulation, the release of neutrophil extracellular traps (NETs) and oxidative burst [13]. Freshly isolated, resting neutrophils were found to be rich in zinc in comparison to other metal ions [14]. Moreover, zinc concentration in neutrophils is correlated with plasma zinc status in patients suffering from sickle cell disease [15].

Recent research provides evidence indicating that zinc levels are correlated with the intensity of innate immunity responses, in particular NETs release. The first reports regarding the impact of zinc on NETs release date to 2009, when Urban et al. reported that zinc inhibits the antifungal activity of NETs. It was also revealed that the presence of calprotectin, zinc-binding protein, in NETs structures is essential for NETs antifungal activity against *Candida albicans* [16]. The indispensability of zinc for the antihyphal activity of calprotectin in NETs structures has also been investigated with the use of *Aspergillus fumigatus* and *Aspergillus nidulans* [17,18]. Interestingly, Ganatra et al. studied NETs release induced by the peritoneal fluid of mice suffering from sepsis [19]. However, all the above reports concern solely NETs induced by fungi or with polymicrobial sepsis. We still lack information about other stimulators, engaging other pathways of NETs release. Further comprehensive research regarding the impact of zinc on the process of NETs formation is desirable.

There are contradictory reports about zinc’s impact on neutrophil phagocytosis. Zinc supplementation was reported to enhance [20] as well as to decrease [21] phagocytosis. In view of these contradictory findings, the impact of zinc ions on neutrophils’ ability to phagocyte remains poorly understood and needs further study. Moreover, we do not know how zinc impacts the degranulation of neutrophil azurophilic granules.

There is also a limited number of studies on the general potential impact of zinc on neutrophils and their functions. In view of this fact, we decided to consistently explore the influence of zinc on the basic defense mechanisms of these cells. Specifically, the aim of our study was to extensively investigate the isolated role of zinc as well as the impact of zinc supplementation and deficiency on phagocytosis, degranulation, oxidative burst and NETs release in various experimental setups. An in-depth analysis of these issues may contribute to a better understanding of how zinc levels affect the innate immune response.

## 2. Materials and Methods

### 2.1. Animals and Feeding

8–12-week-old C57BL/6 mice were purchased from the Animal House of the Polish Academy of Sciences, Medical Research Center. Mice were maintained and bred according to the II Local Ethics Committee at the Warsaw University of Life Sciences, Poland (reference number: WAW2/173/2018) with sawdust in zinc-free plastic cages. Custom prepared diet was purchased from Research Diets, Inc. (New Brunswick, NJ, USA). Mice were fed low-zinc (0.5 mg Zn/kg) content diet and high-zinc (180 mg Zn/kg) content diet for 6 weeks [22,23]. Control group mice received a wholesome diet (containing 30 mg Zn/kg).

### 2.2. Neutrophils and Cell Culture Preparation

Buffy coats or peripheral citrated venous blood were obtained from the regional blood donation center. All donors were free of any acute inflammation. Diluted cell mass was layered on Histopaque 1077 (Ref. no: 10771, Sigma Aldrich, St. Louis, MO, USA) and centrifuged for 30 min at 400 g as described previously [24]. Briefly, a cell pellet containing red blood cells and neutrophils was gently mixed with 1% polyvinyl alcohol. After this, residual red blood cells were lysed with water. Granulocytes were washed and resuspended in RPMI-1640 without phenol red medium with 10 mM HEPES (Ref. no: 17-737E, Lonza, Basel, Switzerland).

The HL-60 cell line was differentiated into granulocyte-like cells with 70 mM dimethylformamide (DMF) for 5 days, as described previously [25].

Whenever indicated, cells were incubated with zinc for 30 min at 37 °C, 5% CO_2_. Based on the optimization experiments (data not shown), a concentration of 10 µM zinc ions was used. Untreated cells served as control.

### 2.3. Murine Neutrophils Preparation

Mice were sacrificed, and blood was collected by cardiac puncture. Serum zinc concentration was analyzed with a zinc assay kit (Ref. no: MAK032, Sigma-Aldrich, St. Louis, MO, USA) according to the manufacturer’s instructions. Additionally, murine tibias, femurs, hips, and humeri were collected. Next, bone marrow from the bones was flushed with HBSS with phenol red supplemented with 0.5% fetal bovine serum and 20 mM HEPES with the use of a 26-gauge needle and passed through a 70-μm cell strainer. After this, lysing buffer (Ref. no: 555899, BD Pharm Lyse, San Jose, CA, USA) was used to remove the remaining red blood cells. Next, cells were layered on Percoll (Ref. no: P1644, Sigma-Aldrich, St. Louis, MO, USA) and centrifuged for 30 min. Finally, cells were resuspended in RPMI-1640 without phenol red with 10 mM HEPES.

### 2.4. NETs Release

Cells (5 × 10^4^ cells/well) were seeded into 24-well plates and left to settle. Next, NETs release was stimulated for 3 h at 37 °C, 5% CO_2_ with 100 nM phorbol 12-myristate 13-acetate (PMA, Ref. no: P1585, Sigma-Aldrich, St. Louis, MO, USA), 4 µM calcium ionophore A23187 (CI, Ref. no: C7522, Sigma-Aldrich, St. Louis, MO, USA), 2.5 µM platelet-activating factor (PAF, Ref. no: 60900, Cayman Chemicals, Ann Arbor, MI, USA), 2 µg/mL lipopolysaccharide (LPS) isolated from *E. coli* (Ref. no: L2755, Sigma-Aldrich, St. Louis, MO, USA) or 10 µg/mL LPS isolated from *P. aeruginosa* (Ref. no: L9143, Sigma-Aldrich, St. Louis, MO, USA). After this, extracellular DNA release was quantified by fluorometry with the use of FLUOstar Omega plate reader (BMG Labtech, Ortenberg, Germany) as described previously [26].

To visualize NETs release, neutrophils were seeded into Lab-Tek chamber cover slides at a density of 2.5 × 10^4^ cells/well and stimulated to release NETs. Next, cells were fixed, permeabilized with 0.1% Triton X and stained overnight at 4 °C with anti-myeloperoxidase-FITC monoclonal antibody (1:500, Abcam ab11729, Cambridge, UK) or anti-neutrophil elastase antibody (1:100, Abcam ab21595, Cambridge, UK). If needed, secondary antibodies conjugated with FITC (1 h, room temperature (RT) 1:2000, Abcam ab6717, Cambridge, UK) were added. DNA was counterstained with 1 μM SYTOX Orange (Life Technologies, Carlsbad, CA, USA) or 5 μg/mL Hoechst 33342 (Abcam ab228551, Cambridge, UK). NETs release was analyzed with the use of an inverted fluorescent microscope Leica DMi8 (Leica, Wetzlar, Germany) equipped with 40× and 10× magnification objectives.

The following concentrations of stimulators of NETs release were applied in experiments with murine neutrophils: 10 µM PAF, 4 µM CI, 40 µg/mL LPS isolated from *P. aeruginosa* and 20 µg/mL LPS isolated from *E. coli*.

### 2.5. Phagocytosis

3 × 10^5^ neutrophils per sample were incubated with 25 µg of fluorescently labeled *E. coli* bioparticles (Thermo Fisher Scientific, Waltham, MA, USA) for 30 min at 37 °C, 5% CO_2_. After this, trypan blue was added to quench extracellular fluorescence and cells were washed with PBS. The percentage of FITC-positive cells was analyzed by cytometry with the use of a BD LSRFortessa flow cytometer (BD Biosciences, San Jose, CA, USA).

### 2.6. Degranulation

To analyze neutrophils degranulation, 5 × 10^5^ of human cells were primed with 25 ng/mL of granulocyte-macrophage colony-stimulating factor (GM-CSF; Ref. no: 300-03, Peprotech, London, UK) for 15 min, incubated with 5 µg/mL cytochalasin B from *Drechslera dematioidea* (Ref. no: C6267, Sigma-Aldrich, St. Louis, MO, USA) for 5 min, followed by incubation with 10 nM of human C5a (Ref. no: HC2101, Hycult Biotech, Uden, The Netherlands) for 15 min. After this, the reaction was stopped with ice-cold PBS. The supernatant was discarded, and cells were labeled with anti-CD63 Pacific Blue antibodies (Ref. no: 353012, BioLegend, San Diego, CA, USA). CD63 expression on the cell surface was analyzed with BD LSR Fortessa flow cytometer.

In the experiments with murine neutrophils, the following concentrations were used: 100 ng/mL murine GM-CSF (Ref. no: 415-ML, R&D Systems, Minneapolis, MN, USA), 5 µg/mL cytochalasin B, 100 nM murine C5a (Ref. no: HC1101, Hycult Biotech, Uden, The Netherlands). Murine neutrophils were labeled with anti-CD63 PE antibodies (Ref. no: 564222, BD Pharmingen, San Jose, CA, USA).

### 2.7. Immunodetection of Citrullinated Histone H3

Neutrophils were treated with 4 µM CI for 30 min at 37 °C, 5% CO_2_. Subsequently, cell pellets were lysed with RIPA buffer supplemented with a protease inhibitor cocktail. Lysates were sonicated and boiled with 5× Lemmli buffer. Equal amounts of protein were separated by SDS–PAGE and transferred to a nitrocellulose membrane. Membranes were blocked with 5% milk and incubated overnight with primary antibody anti-citH3 (ab5103, 1:1000 in 5% milk, 4 °C, Abcam, Cambridge, UK). Next, secondary anti-rabbit antibodies conjugated with HRP (#7074, 1:2000 in 5% milk, 1 h, RT, Cell Signaling, Danvers, MA, USA) were added. Anti-ACTB antibodies conjugated with HRP (#A3854, 1:50,000 in 5% milk, 0.5 h incubation at RT, Sigma-Aldrich, St. Louis, MO, USA) served as loading controls.

### 2.8. Oxidative Burst Measurement

To analyze the oxidative burst by fluorometry, neutrophils (1 × 10^5^/well) were seeded into black 96-well plates and loaded with 4 µg/mL dihydrorhodamine (DHR, Ref. no: D23806, Thermo Fisher Scientific, Waltham, MA, USA) 123. After this, cells were stimulated with PMA, and the fluorescence intensity was measured as described previously [26].

For the chemiluminescence assay, cells (5 × 10^4^/well) were seeded into 96-well white plates with 2.5 mg/mL glucose, 2.5 mg/mL bovine serum albumin and 10 μM luminol (Ref. no: A8511, Sigma-Aldrich, St. Louis, MO, USA). After this, neutrophils were stimulated to release reactive oxygen species (ROS) with 100 nM PMA. Chemiluminescence was measured every 6 min for 1 h with FLUOstar Omega plate reader.

Nitro blue tetrazolium (NBT) reduction assay was performed as described previously [27]. Briefly, 2.5 × 10^4^/well cells were seeded into Lab-Tek chamber cover slides and loaded with 2 mg/mL nitroblue tetrazolium chloride (Ref. no: J60230, Alfa Esar, Haverhill, MA, USA). After this, the oxidative burst was stimulated with 100 nM PMA and the percentage of blue cells containing formazan crystals was counted by light microscopy.

### 2.9. Neutrophil Elastase Activity Assay

To analyze neutrophil elastase (NE) activity, neutrophils (11.25 × 10^4^/well) were seeded into 24-well plates and left to settle for 30 min at 37 °C, 5% CO_2_. Next, NETs release was stimulated with 100 nM PMA for 3 h. Unbound NE was washed away, and NETs were digested with 10 U/mL DNase. After the reaction was stopped, the plates were centrifuged, the supernatant was collected, and 250 µM elastase substrate (Ref. no: M4765, Sigma-Aldrich, St. Louis, MO, USA) was added. FLUOstar Omega plate reader was used to measure the absorbance of 4-nitroaniline product at wavelength 405 nm.

### 2.10. Bacterial Killing Assay

To analyze extracellular bacterial killing, 2 × 10^4^ neutrophils per well were seeded into 48-well plates and left to settle. After this, *E. coli* (ATCC^®^ 25922™, the multiplicity of infection 10 *E. coli*: 1 neutrophils) were added. Plates were centrifuged and incubated for 3 h at 37 °C, 5% CO_2_. Next, 10 U/mL DNase was added, and serial dilutions of the bacterial suspension were plated on LB agar plates in duplicates. After overnight incubation at 37 °C, the number of colony-forming units (CFU) of bacterial colonies were analyzed.

### 2.11. Statistical Analysis

Statistical analysis was performed using GraphPad Prism 8 (GraphPad Software, La Jolla, CA, USA). The normality of distribution was assessed by Shapiro–Wilk test. All data were analyzed with one-way or two-way ANOVA, followed by appropriate post hoc tests as specified in the text. Unless stated otherwise, all results are presented as mean + standard error of the mean. Results were considered statistically significant at *p* ≤ 0.05.

## 3. Results

### 3.1. Zinc Inhibits NETs Release

**Figure 1:**
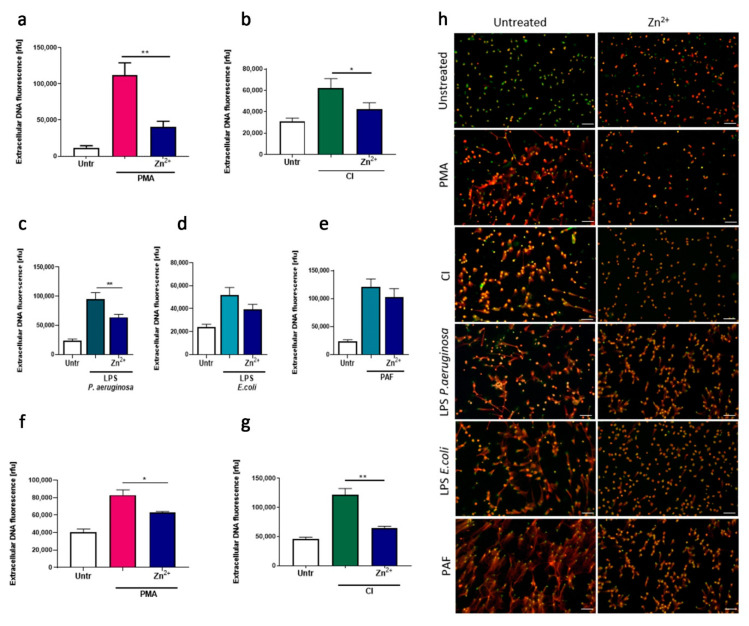
Neutrophils incubated with zinc release less neutrophil extracellular traps (NETs). (**a**–**e**,**h**) Neutrophils were isolated from peripheral blood of healthy donors. (**f**,**g**) HL-60 cells were differentiated with dimethylformamide into granulocyte-like cells. (**a**–**e**,**h**) Cells were incubated with or without 10 µM zinc and stimulated with (**a**,**f**,**h**) 100 nM phorbol 12-myristate 13-acetate (PMA), (**b**,**g**,**h**) 4 µM calcium ionophore A23187 (CI), (**c**,**h**) 10 µg/mL lipopolysaccharide isolated from *P. aeruginosa* (LPS PA), (**d**,**h**) 2 µg/mL lipopolysaccharide isolated from *E. coli* (LPS *E. coli*) or (**e**,**h**) 2.5 µM platelet activating factor (PAF). NETs release was analyzed microscopically and released DNA was quantified fluorometrically. Data are shown as means + SEM and were analyzed by one-way ANOVA with post hoc Dunn’s test (**a**,**b**), Holm–Sidak’s test (**g**), Tukey’s test (**f**), or Sidak’s test (**c**–**e**); (**a**–**c**) *n* = 6, (**f**,**g**) *n* = 3, * (*p* ≤ 0.05), ** (*p* ≤ 0.01). MPO myeloperoxidase; (**h**) scale bar = 50 um.

Firstly, we analyzed the influence of zinc ions on lytic NETs released by human neutrophils. Pretreatment of cells with zinc showed strong inhibition of NETs release after stimulation with PMA, CI, and LPS isolated from *P. aeruginosa* (Figure 1a–c,h). Furthermore, using fluorescence microscopy, we could observe the same inhibitory effect of zinc on NETs release stimulated by LPS isolated from *E. coli* and PAF, although quantitative analysis by fluorometry showed only a decreasing trend without statistical significance (Figure 1d,e,h). Furthermore, we aimed to corroborate our observations using a cell line model. To that end, we differentiated HL-60 cells with DMF and stimulated them to release NETs by PMA and CI. In both settings, zinc inhibited NETs release (Figure 1f,g).

### 3.2. Zinc Effectively Inhibits Citrullination of Histone H3 but Does Not Affect ROS Release and NE Activity

**Figure 2:**
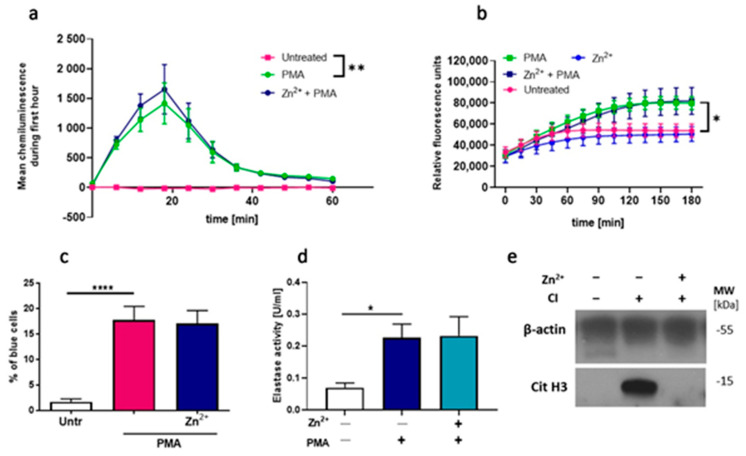
Zinc does not affect reactive oxygen species (ROS) release and neutrophil elastase (NE) activity but inhibits the citrullination of histone H3. Neutrophils were incubated with or without 10 µM zinc and (**a**) 10 μM luminol, (**b**) 4 μg/mL dihydrorhodamine (DHR) 123 or (**c**) 2 mg/mL nitroblue tetrazolium (NBT). Next, cells were stimulated with (**a**–**d**) 100 nM PMA or (**e**) 4 µM CI. Post stimulation, (**a**) chemiluminescence was measured; (**b**) fluorescence was analyzed; (**c**) the percentage of cells containing deposits of formazan was counted; (**d**) extracellular NE was washed away, NETs were digested to release NE from NETs, and NE activity was measured (**e**) immunodetection of citrullinated histone H3 was performed. One-way ANOVA with post hoc (**a**,**c**) Tukey’s test; (**d**) Dunnett’s test. (**b**) Two-way ANOVA with post hoc Dunnett’s test. The means (**a**,**b**) ± SEM; (**c**,**d**) + SEM are shown; (**a**) *n* = 3, (**b**) *n* = 6, (**c**) *n* = 9, (**d**) *n* = 7, (**e**) one representative out of 3 experiments from three different blood donors is shown; * (*p* ≤ 0.05), ** (*p* ≤ 0.01), **** (*p* ≤ 0.0001).

Our further step was to answer the question of which mechanism is responsible for zinc-mediated inhibition of NETs release. It was suggested that two processes are of critical importance for NETs formation: generation of ROS by nicotinamide adenine dinucleotide phosphate (NADPH) oxidase and histone citrullination [28]. First, we examined whether NADPH oxidase-dependent ROS production, one of the critical processes in NETs formation [29], is affected by zinc. Accordingly, we used three different attempts, and we analyzed the oxidative burst in neutrophils by performing luminol-amplified chemiluminescence, DHR 123 oxidation and NBT reduction assays. PMA, which is a strong activator of protein kinase C (PKC), which further activates NADPH oxidase, leading to ROS formation [30], was used as a stimulator. Obtained results revealed that zinc did not alter ROS production (Figure 2a–c). Next, we targeted the pathway downstream ROS release and analyzed the impact of zinc on neutrophil elastase (NE) activity. Our results showed that zinc does not affect NE activity (Figure 2d). In addition, we decided to verify if zinc affects the NADPH-oxidase-independent pathway via calcium-activated potassium (SK3) channel activation and histone citrullination. To this end, we stimulated cells with calcium ionophore, which was already shown to stimulate release NETs through SK3 channels activation [31], and performed immunodetection of citrullinated histone H3. We did not evaluate histone citrullination under the influence of other stimulators than CI since it has been previously proved that hypercitrullination of histone H3 is only observed in calcium ionophore-mediated NETosis [25,31]. Our observations indicate that zinc effectively inhibits the citrullination of histone H3 (Figure 2e).

### 3.3. Zinc Inhibits Degranulation and Limits the Number of bactEria, but Does Not Affect Phagocytosis

**Figure 3:**
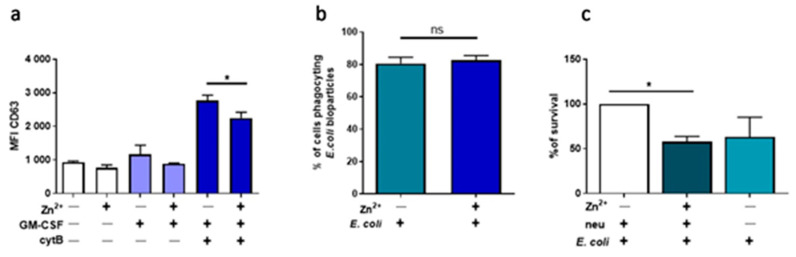
Zinc affects degranulation and the number of bacteria but does not alter phagocytosis. Neutrophils were incubated with or without 10 µM zinc and (**a**) primed with 25 ng/mL GM-CSF, incubated with 5 µg/mL cytochalasin B, 10 nM C5a, immunolabeled and analyzed by flow cytometry; (**b**) incubated with *E. coli* bioparticles and analysis of the percentage of cells phagocyting was analyzed by flow cytometry; (**c**) incubated with alive *E. coli* at the multiplicity of infection 10:1 (*E. coli*: neutrophils) for 3 h and the number of colonies of survived bacteria from the collected supernatants were counted. One-way ANOVA with post hoc (**a**) Sidak’s test, (**b**) Holm–Sidak’s test, (**c**) Kruskal–Wallis test. The means + SEM are shown; (**a**) *n* = 3, (**b**) *n* = 6, (**c**) *n* = 4; * (*p* ≤ 0.05).

Having shown that zinc is a strong inhibitor of NETs release, we wondered if it could also affect other neutrophils’ functions, including degranulation, phagocytosis, and bacterial killing. In order to examine the impact of zinc on neutrophils’ degranulation, we determined the mobilization of azurophilic granules by analyzing CD63 expression on the cell surface. Cytometric analysis showed that in vitro treatment of cells with zinc leads to a decrease of CD63 expression (Figure 3a). To analyze the effect of zinc on the phagocytic abilities of neutrophils, cells were incubated with *E. coli* bioparticles conjugated with FITC. Analysis of FITC-positive cells revealed no differences between the studied groups. Furthermore, to evaluate the impact of zinc on the bactericidal properties of neutrophils, we performed a bacterial killing assay. To this end, cells were preincubated with zinc, and alive *E. coli* was added. We observed that the preincubation of cells with zinc decreased the number of bacteria (Figure 3c).

### 3.4. Zinc Inhibits NETs Release in Neutrophils Isolated from Mice, but Does Not Affect ROS Production, Phagocytosis nor Degranulation

**Figure 4:**
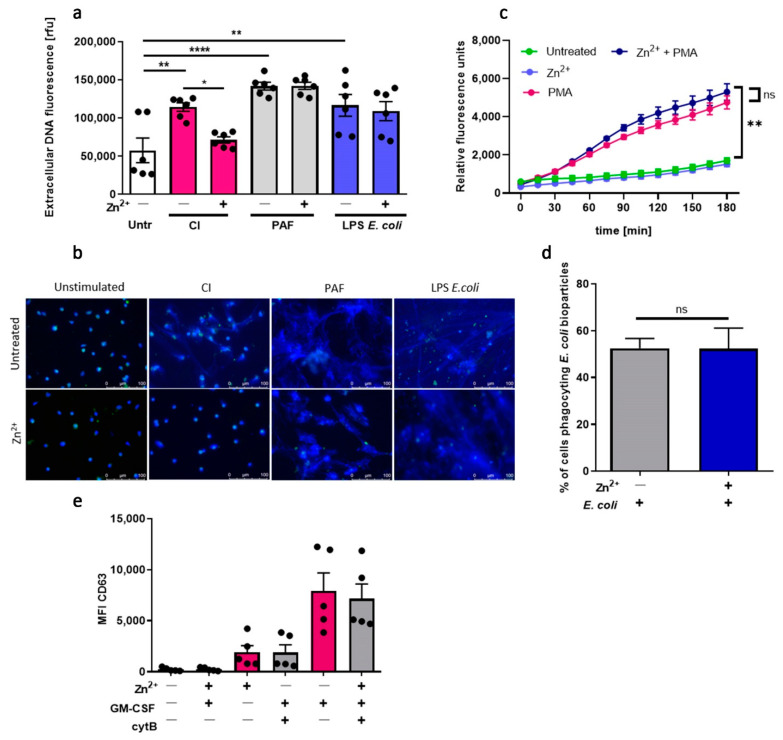
Zinc inhibits NETs release in neutrophils isolated from mice. Mice were sacrificed, and bone marrow neutrophils were isolated. (**a**,**b**) Neutrophils were stimulated to release NETs with 4 µM CI, 10 µM PAF or 20 µg/mL LPS *E. coli*. NETs release was (**a**) measured fluorometrically and (**b**) analyzed by fluorescent microscopy. (**c**) Neutrophils were incubated with 4 µg/mL DHR 123 and stimulated with 100 nM PMA. ROS production was measured fluorometrically. (**d**) Cells were incubated with *E. coli* bioparticles, and the percentage of cells phagocyting bacteria was analyzed by flow cytometry. (**e**) Neutrophils were primed with GM-CSF, incubated with cytochalasin B, C5a and labeled with anti-CD63. Data are shown as means (**a**,**d**,**e**) + SEM; (c) ± SEM; (**a**,**e**) with individual values. One-way ANOVA with post hoc (**a**) Bonferroni’s test, (**d**) Holm–Sidak’s test; (**e**) Dunn’s test. Two-way ANOVA with post hoc (**c**) Dunnett’s test. (**a**,**b**) *n* = 6, (**c**) *n* = 4, (**d**) *n* = 7, (**e**) *n* = 5; * (*p* ≤ 0.05), ** (*p* ≤ 0.01), **** (*p* ≤ 0.0001). NE: neutrophil elastase.

As a complementary approach, we investigated the impact of zinc on the functions of murine neutrophils isolated from mice. To that end, we stimulated murine neutrophils with CI, PAF and LPS isolated from *E. coli* to stimulate NETs release. We found that zinc selectively inhibits NETs release stimulated by CI (Figure 4a,b). Pretreatment of murine neutrophils with zinc, similarly to human neutrophils, did not affect ROS production (Figure 4c), phagocytosis (Figure 4d), nor degranulation (Figure 4e).

### 3.5. Low Zinc Diet Increases NETs Release and Neutrophils’ Degranulation

**Figure 5:**
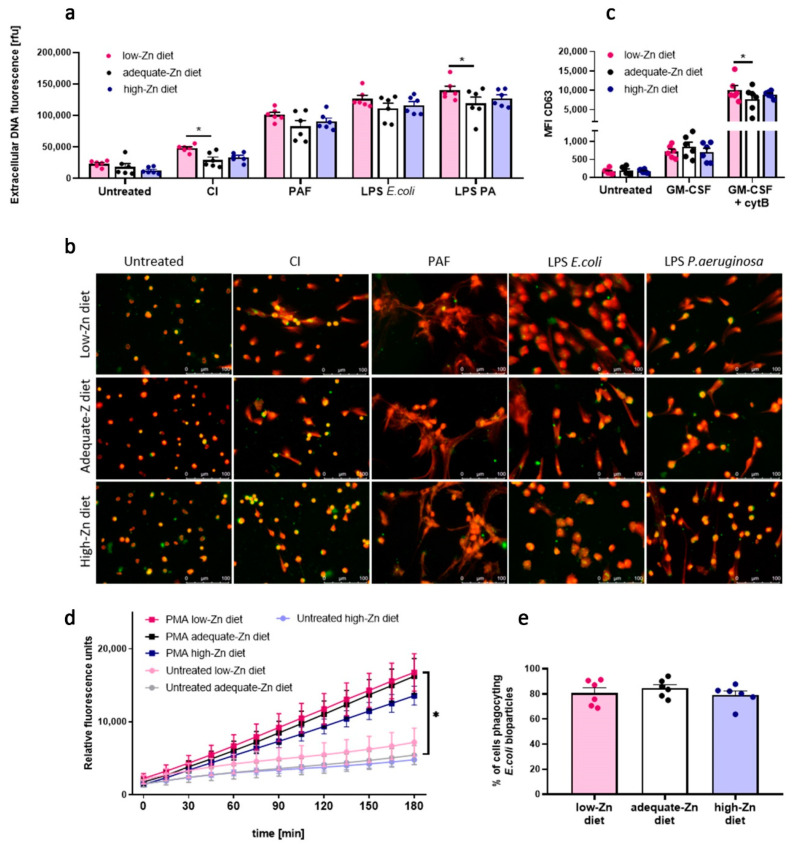
Neutrophils of mice fed low-zinc content diet present decreased NETs release. Mice fed with a low-zinc and high-zinc content diet were sacrificed, and bone marrow neutrophils were isolated. (**a**,**b**) NETs release was stimulated with 4 µM CI, 10 µM PAF, 20 µg/mL LPS *E. coli*, 40 µg/mL LPS *P. aeruginosa*. Next, NETs release was analyzed by (**a**) fluorometry and (**b**) fluorescent microscopy. (**c**) Degranulation of CD63 was analyzed by flow cytometry. (**d**) ROS production was analyzed by fluorometry. (**e**) Percentage of phagocyting cells was analyzed by flow cytometry. (**a**,**c**,**d**) Two-way ANOVA with post hoc Dunnett’s test. (**e**) One-way ANOVA with post hoc Dunnett’s test. The means (**a**,**c**,**e**) + SEM; (**d**) ± SEM; (**a**,**c**,**e**) with individual values are shown; *n* = 6; * (*p* ≤ 0.05). NE: neutrophil elastase.

To evaluate the biological relevance of our findings and to further study the role of zinc disturbances on innate immunity, we fed two separate groups of mice with low-zinc and with high-zinc content diets, respectively. Mice fed with a wholesome, adequate diet constituted the control group. After the feeding time of six weeks, differences in zinc concentration in mice serum between groups could be observed (*p* ≤ 0.05) (Supplementary material Figure S1a). Moreover, mice fed with a low-zinc diet weighted less than mice fed with a control diet (*p* ≤ 0.01) (Supplementary material Figure S1b). We found that neutrophils isolated from mice fed with a low-zinc content diet revealed a significantly higher release of NETs compared to the control group when NETs release was stimulated with CI and LPS isolated from *P. aeruginosa*. A similar trend, although not statistical, was observed when NETs release was induced with PAF and LPS isolated from *E. coli* (Figure 5a,b). Moreover, neutrophils isolated from mice fed a low-zinc content diet showed a higher expression of CD63 on the cell surface than neutrophils of mice fed with a wholesome diet (Figure 5c). Fluorometric analysis of DHR 123 oxidation showed no impact of low-zinc and high-zinc content diet on ROS production (Figure 5d). Regarding neutrophils’ ability to phagocyte, no differences between groups were observed (Figure 5e).

## 4. Discussion

In this study, we investigated the impact of zinc on the functions of neutrophils isolated from healthy humans and mice. We analyzed how zinc affects neutrophils functions, in particular phagocytosis, degranulation, oxidative burst and release of NETs. Moreover, we generated a model of zinc deficiency and studied the influence of zinc supplementation on innate immunity in mice. Overall, our results demonstrate that zinc modulates neutrophils functions by affecting NETs release and neutrophil degranulation.

As described by Scott and Bradwell in 1983, zinc in serum is mostly bound to the albumin—a major zinc-binding protein, whereas a small fraction of 2% of total zinc in the human body is unbound [32,33]. A drop in zinc level in plasma was observed in patients suffering from sepsis by Besecker et al. [34]. Zinc deficiency was found to be associated with increased mortality in a murine model of sepsis [35]. Importantly, prophylactic zinc supplementation increased survival in mice suffering from sepsis and reduced the number of bacteria in the peritoneal fluid, lungs, blood, and spleen in mice [36]. All above-mentioned reports indicate that zinc homeostasis is crucial for a proper innate immune response.

Our findings demonstrate that zinc inhibits NETs release stimulated by various stimuli under in vitro conditions and this effect is biologically relevant in various species, observed both in neutrophils isolated from healthy humans and from mice. Our results are in line with Wessels et al., who observed the same inhibitory effect of zinc on NETs release in humans, but with the use of a different stimulator—N-formylmethionyl-leucyl-phenylalanine (fMLP). Moreover, this group also had the same observation in mice injected with zinc [37]. Interestingly, Ganatra et al. reported contrary observations where zinc supplemented by injection caused an increase in NETs release, and moreover, zinc did not affect NETs efficacy to kill bacteria [19]. In our study, mice fed with a high-zinc content diet did not differ in the release of NETs from mice fed a wholesome diet. Given that in other reports, zinc was supplemented by injection, we assume that in our study, mice fed a high-zinc content diet did not reach sufficiently high concentrations of zinc, which could induce such a strong systemic impact as in Ganatra et al.

Zinc necessity for NETs release was reported by Hasan et al., who indicated that activation of neutrophils with PMA triggers an intracellular rise in free zinc concentration. Moreover, they showed that zinc sequestration by a membrane permeable chelator *N,N,N′,N′-*tetrakis(2-pyridinylmethyl)-1,2-ethanediamine (TPEN) reduced the release of NETs [38]. In our study, the capability of releasing NETs by neutrophils of mice fed a low-zinc content diet was not compromised. On the contrary, zinc deficiency caused an even higher release of NETs by neutrophils when compared to the control group. These results demonstrate that proper levels of zinc are essential for the precise functioning of the innate immune system, and both zinc overload and zinc deficiency may modulate granulocytic functions.

Beyond NETs release investigations, there are contradictory reports about zinc’s influence on neutrophils’ ability to produce ROS. Zinc was reported to inhibit ROS release [3,39] as well as to increase the oxidative burst in neutrophils [40,41]. The oxidative burst in NETs release was investigated by Hasan et al., who reported that zinc had no effect on PKC activation nor oxidative burst. The authors suggested that zinc is necessary for NETs formation, however downstream ROS production [38]. This is in accordance with our study, as we did not observe any effect of zinc on ROS production.

Calcium ionophore is known to stimulate NETs to release through peptidylarginine deiminase 4 (PAD4) activation and histone citrullination [42]. Moreover, Kearney et al. documented that metal ions— zinc, among others—strongly inhibit the calcium-induced activation of PAD4 [43]. For this reason, we decided to investigate if histone citrullination is affected by zinc and performed immunodetection of citrullinated histone H3. Our observations confirm that zinc effectively inhibits the citrullination of histone H3. Accordingly, we presume that zinc inhibits NETs release by affecting histone citrullination when NETs release is stimulated by CI. Regarding the mechanism by which PMA affects NETs release, further studies are warranted to fully explore this issue.

The NETs studies that we present in this paper have some limitations. We would like to underline that our experiments were focused exclusively on lytic NETosis, thus based on our results, we cannot assess if mitochondrial NETs release may be affected by zinc supplementation. This issue could be the subject of further research.

Sunzel et al. observed that granulocytes pretreated with high concentrations of zinc were protected against damage caused by exposure to *Staphylococcus aureus* and suggested that this effect was due to the neutralization of bacteria-derived toxins [44]. In our study, we observed that zinc limited the number of *E. coli* in the presence of neutrophils. Altogether, we suggest that zinc can affect bacteria harmfulness and influence their survival.

In our study, the addition of zinc decreased the intensity of degranulation analyzed as the expression of azurophilic granules’ a marker—CD63—on neutrophils isolated from healthy individuals. This trend could also be observed in murine neutrophils pretreated with zinc in vitro prior to neutrophils’ stimulation. Our findings are in line with Wessels et al., who had the same observations in relation to the expression of CD35, which is a marker of secretory vesicles, in neutrophils pretreated with zinc [37]. Moreover, our results showed that mice fed with a low-zinc content diet presented an increase in CD63 expression. Contrastingly, Hasan et al. reported that zinc sequestration in the cell reduced the expression of CD35, CD66b and Mac-1, markers of neutrophils’ specific granules and secretory vesicles on the cell surface [45]. However, it should be underlined that metal sequestration induced by a chemical chelator can trigger a different effect than metal deficiency caused by a diet. Moreover, zinc deficiency can have a different effect depending on the type of neutrophilic granules. In our study, we saw a difference in degranulation rate between human and murine neutrophils under the influence of zinc. We deduce that murine neutrophils are less susceptible to zinc impact. Maybe if the zinc concentration were higher or the incubation time longer, we would be able to have the same observations as in humans. Nevertheless, our aim was to have the same experimental conditions in all studied models, even though it could limit our findings in a murine model.

Zinc supplementation was reported to enhance phagocytosis in mice and in dairy cows [19,20], whereas zinc deficiency caused a decrease in phagocytosis [45,46]. Contrarily, Yatsuyanagi et al. reported a decrease in phagocytosis levels of neutrophils in response to high doses of zinc in rats [21]. In our experiment, we did not observe any effect of zinc deficiency or zinc supplementation on this process. Our results are in line with Sunzel et al., who also did not report any effect of zinc on phagocytosis [44]. Given that in each of the studies, different species, zinc concentrations and methodology were applied, we suggest that discrepancies between reports can be attributed to differences in experimental setups.

In conclusion, our study demonstrates that excess of zinc or zinc deficiency may cause alterations in the functioning of neutrophils. Accurate zinc concentrations in the body are invaluable to maintain proper functioning of the innate immune response.

## Data Availability

The data presented in this study are available on request from the corresponding author. The data are not publicly available due to ethical and privacy reasons.

## Supplementary Materials

The following are available online at https://www.mdpi.com/2072-6643/13/1/51/s1, Figure S1: Zinc concentration in serum and bodyweight of mice fed low-zinc and high-zinc content diet.
