# Supplementary material for: Zinc Supplementation Modulates NETs Release and Neutrophils’ Degranulation
Source: Nutrients. 2020 Dec 26;13(1):51. doi: 10.3390/nu13010051 (PMC7823768; doi:10.3390/nu13010051)
Supplement: Supplementary file 1 [file nutrients-13-00051-s001.pdf]

Supplementary information

Zinc supplementation modulates NETs release and neutrophils' degranulation

Weronika Kuźmicka[1,2], Aneta Manda-Handzlik[2], Adrianna Cieloch[2], Agnieszka Mroczek[2], Urszula Demkow[2], Małgorzata Wachowska[2], Olga Ciepiela[3]

1 - Postgraduate School of Molecular Medicine, Medical University of Warsaw, Zwirki i Wigury 61 Street, 02-091 Warsaw, Poland

2 - Department of Laboratory Diagnostics and Clinical Immunology of Developmental Age, Medical University of Warsaw, Zwirki i Wigury 63a Street, 02-091 Warsaw, Poland

3 - Department of Laboratory Medicine, Medical University of Warsaw, Banacha 1a Street, 02-097, Warsaw, Poland

Corresponding authors: Olga Ciepiela and Małgorzata Wachowska

e-mail: olga.ciepiela@wum.edu.pl; malgorzata.wachowska@wum.edu.pl

telephone number: 22 317 95 03

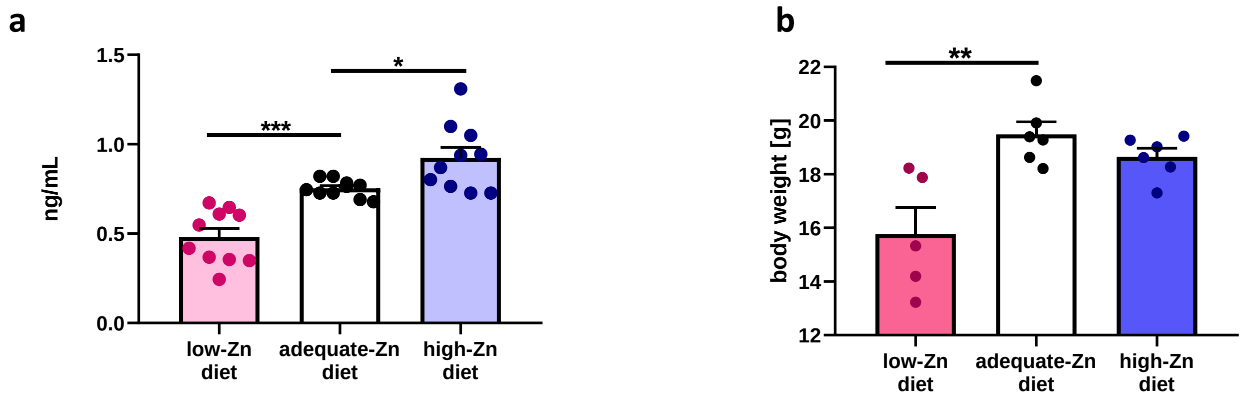

Supplementary Figure 1. **Zinc concentration in serum and body weight of mice fed low-zinc and high-zinc content diet.** (**a**) Mice were sacrificed, and blood was collected by cardiac puncture in order to analyze zinc serum concentration. (**b**) Mice were weighted. Data are shown as means **(a, b)** + SEM and were analyzed by one-way ANOVA with post hoc Dunnett's test; (**a**) n=10, (**b**) n=5 for low-Zn content diet and n=6 for adequate and high-Zn content diet.
